# “I want to live, but …” the desire to live and its physical, psychological, spiritual, and social factors among advanced cancer patients: evidence from the APPROACH study in India

**DOI:** 10.1186/s12904-022-01041-z

**Published:** 2022-08-31

**Authors:** Anjum S. Khan Joad, Arati Hota, Pratima Agarwal, Krimal Patel, Kinjal Patel, Jyotika Puri, Soye Shin

**Affiliations:** 1 Bhagwan Mahaveer Cancer Hospital and Research Centre, Jaipur, India; 2 Program in Health Services and Systems Research, Duke-NUS Medical School, Singapore, Singapore

**Keywords:** Advanced cancer patients, Palliative care, Desire to live, Desire for hastened death, India, Socio-economic status, Pain severity, Psychological distress

## Abstract

**Background:**

The limited access to palliative care resources along with the social stigma around cancer largely explains the poor quality of life (QoL) of Indian advanced cancer patients. As advanced cancer patients with poor QoL often harbour a desire for hastened death (DHD), it is imperative to understand factors affecting DHD, or the desire to live (DTL) among advanced cancer patients in India. We aim to examine the relationship between DTL and physical, psychological, spiritual, and social factors measuring patients’ QoL alongside their awareness of their late cancer stage.

**Methods:**

We surveyed 200 patients from a tertiary cancer hospital in India to collect their DTL, awareness of cancer stage, demographic characteristics, and standardized measures for patients’ QoL. We used a linear probability regression model to quantify the association between these factors and patients’ DTL among the final sample of 192 patients with no missing information for the variables of interest.

**Results:**

Among the various domains affecting cancer patients’ QoL, we found that the pain severity score (ranging from 0 to 10) and psychological distress score (ranging from 0 to 42) are negatively associated with the DTL. One point increase in each score reduced the DTL by 2.2% (*p* < 0.05) and 0.7% (*p* < 0.05), respectively. Our results also showed that patients whose perceived socio-economic status (SES) is poor have a 16% (*p* < 0.05) lower probability of DTL, compared to those with higher SES (lower middle class, upper middle class, and wealthy). Controlling for caste, religion, gender, age, marital status and years of education, we found psychological distress is statistically higher among patients belonging to this bottom SES.

**Conclusions:**

We found that pain severity, psychological distress and perceived low SES are negatively associated with the desire to live in advanced cancer patients. Future research should focus on developing interventions to improve physical pain and psychological distress, particularly for patients who are socially and economically disadvantaged.

## Introduction

*He who has a “why” to live can deal with almost any “how”. –Nietzsche*

([1] p.IX)

According to Global Cancer Data from GLOBOCAN 2018 [2], an interactive web platform that consolidates worldwide cancer statistics, in India, of 2.2 million cancer patients, 784,000 die annually. This number has doubled since 1990 [3]. The age-standardised mortality: incidence ratio for India is 0.68, which is higher than that of very high human development index (HDI) countries (0.38) and high HDI countries (0.57) [4]. This is a big concern as India has a relatively younger population than those countries and hence is expected to present lower age-adjusted mortality indices. Part of this mismatch can be attributed to better screening practices in high HDI nations, but a large part of it is because of inequitable access to quality cancer care, lack of affordability in care and delayed diagnosis of cancer due to stigma related to the illness and a lack of awareness of its symptoms [5].

Various myths and misconceptions regarding cancer cause delays in patients seeking medical care. A survey of 95 cancer patients in Delhi showed that one third of the patients believed malignancies can be detected in early stages and that they are curable [6]. Most of the surveyed patients were reported to hold fatalistic views about the outcome of cancer and 60% felt that their family and society were discriminating against them because of their cancer diagnosis. As a result, the average time taken by patients to report to a doctor after suspecting their disease was 2 years [6]. By that time, their cancer has often developed to advanced stages [7–9]. Moreover, limited access to palliative care and comprehensive cancer treatment services contributes to the high mortality rate from cancer in India. Less than 4% of patients with serious health-related suffering have access to opioid morphine-equivalents [10]. Also, more than 75% of cancer expenditures in India are covered out-of-pocket, which indicates that access to affordable and equitable cancer care in India still remains a major challenge [11].

Combined with the prevalence of advanced stage cancer at the first diagnosis, the limited access to cancer and pain treatment resources along with the social stigma around cancer largely explains poor quality of life (QoL) of Indian advanced cancer patients. There is comprehensive literature showing that cancer patients with poor QoL often harbour a desire for hastened death (DHD) [12–15]. The frequency of reporting DHD among advanced cancer patients is noticeably higher than that among patients in nonpalliative stages of cancer [16], which is a significant concern for healthcare professionals and stakeholders [17]. Despite the urgency of the issue, there is limited research on factors that may increase DHD in advanced cancer patients, particularly in the context of India.

The aim of the study is to fill this knowledge gap by identifying contributors to the desire to live (DTL), the other side of DHD, with advanced cancer patients in a tertiary cancer centre in Jaipur, India. Previous studies, mostly conducted in developed countries, provided evidence that DTL (or, DHD) was associated with various physical, psychological, spiritual, social or interpersonal and spiritual factors. For instance, one study reported that as many as 77% terminally ill patients desired to hasten death because of experiencing moderate to severe physical pain [18]. The psychological and emotional distress of cancer patients is also negatively associated with their DTL. Individuals with advanced-stage cancer reported persistent depressive symptoms more frequently [19] and both depression and hopelessness independently predicted DHD of metastatic cancer patients [20]. In particular, patients with low socio-economic status (SES) tend to reveal a higher prevalence of depression [21]. In addition, experiencing low social support hinders people’s ability to find meaning in their lives, which contributes to high DHD. Lastly, spiritual well-being or belief in a higher power has been established as an important protective factor that keeps depression and hopelessness at bay and builds some tolerance to physical symptoms [22].

Guided by the evidence, we collected patients’ awareness of their advanced cancer along with multiple indexes measuring their QoL, in terms of physical (e.g., pain severity), psychological (e.g., self-blame), spiritual (e.g., faith), and social support (e.g., social stigma) dimensions, and examined the association of DTL and these variables. We hypothesize that patients who suffer from severe physical pain, a high level of anxiety & depression, and have low spiritual well-being and social/family support are likely to have a lower DTL. We also hypothesize that patients from a socially and economically disadvantaged group are likely to present a lower DTL, holding other factors constant.

Focusing on the second hypothesis, we further seek to investigate whether there are significant differences in the QoL factors, by patients’ perceived socio-economic strata. We hypothesize that patients who reported to have lower SES would experience higher social stigma, psychological distress, and lower interpersonal support. Testing this hypothesis is especially important in a country as diverse as India with multifaceted segmentation based on caste, class, religion, regional economic advancement, and language. For instance, at the regional level, development of palliative care and pain management services is uneven in that there is greater provision of the services in the southern parts than the north [23]. The unequal access to these services is consistent with regional economic gaps that five states of south India account for about 30% of India’s Gross Domestic Product (GDP) while eight states of north India account for only 2.8% [24]. We expect QoL disparities by SES exist at the household/individual level as well.

To the authors’ best knowledge, there are no prior studies comprehensively examining the association between DTL and various QoL factors among advanced cancer patients in India. Previous work with cancer patients in India has rather focused on the prevalence of suicidal ideation [14, 15]. We expect findings from this study to deepen our understanding of various factors that may affect DTL in a regional cancer centre in India. This would provide insights into advanced cancer patient care not only in India but in many other developing countries where palliative and pain care services are limited.

## Methods

### Setting and participants

This study was part of a multi-country cross-sectional survey titled “Asian Patient Perspectives Regarding Oncology Awareness, Care and Health (APPROACH)” to assess gaps in care received by advanced cancer patients seeking care at major public hospitals in low- and middle- income countries in Asia. The site in the study is Bhagwan Mahaveer Cancer Hospital and Research Centre (BMCHRC) in Jaipur, India. Established in 1997, BMCHRC is a specialty hospital offering cancer prevention, treatment, research, and education-related services, especially for economically-disadvantaged groups of the population by subsidizing treatments or in some cases, providing it free of cost. Approximately 14,000 cancer patients visit the hospital for treatment from Rajasthan (state where Jaipur is located) and neighbouring states every year. A large proportion or the patients are from low socio-economic strata and present with advanced stages of cancer when they first consult with physicians.

We recruited inpatients and outpatients from the departments of medical oncology and radiotherapy and palliative care from 7th March 2017 to 16th August 2018. Inclusion criteria was patients who were over the age of 21, diagnosed with stage 4 solid cancer, and aware of cancer diagnosis (not necessarily its stage). Only patients with solid cancer were included because those who were diagnosed with cancers like lymphoma or leukemia progress differently and have a different staging system which would create inconsistencies in analysis and interpretation.

The study was approved by the Institutional Ethics Committee of Bhagwan Mahaveer Cancer Hospital & Research Centre (Ref No: BMH/ 2016/89) and the National University of Singapore (Ref No: B-15-319).

**Fig. 1:**
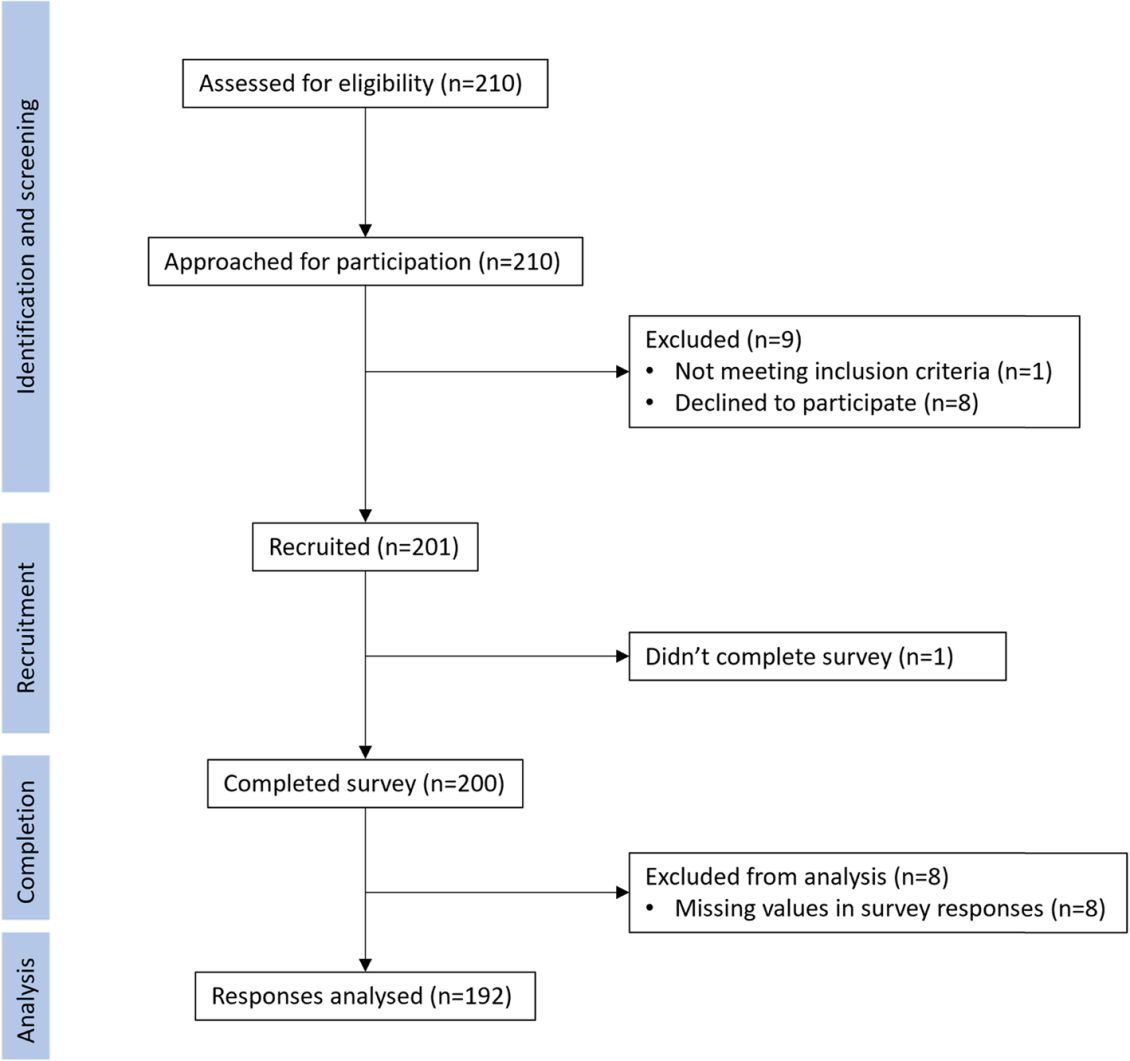
Patient recruitment log

Figure 1 presents the patient recruitment diagram. Targeting a sample of 200 patients, the research team screened 210 medical records for the eligibility criteria and identified 209 eligible patients. Among these eligible patients that our trained interviewers approached, 8 patients refused to participate in the study. From 201 patients who consented to the study, 200 completed the interview, which results in a response rate of 99.5%.

### Survey questionnaire

In consultation with oncologists, we developed a questionnaire and validated instruments to measure patients’ QoL in physical, psychological, spiritual, and social domains. We also collected patients’ demographic information that includes gender, age, education level, marital status, SES, caste (if any), religion, ethnicity as well as their awareness of their advanced cancer stage. All questions in English were translated by professional translators into Hindi, which is commonly spoken at the site, according to a strict translation protocol for interview, and patients’ answers were translated back to English. Cross-validation between the original and back translated versions were made to ensure the consistency. More details about the dependent and independent variables (predictors) of interest are discussed below.

#### Outcome variable: DTL

In the survey, we asked them a single question enquiring if they ever wished their life would end sooner. They could answer it with yes, no, or not sure. The source of this question was the first item of the Desire for Death Rating Scale [18], which underwent pilot testing on 10 patients who met the eligibility criteria before being included in the survey. The reason for choosing a single-item measure as opposed to a multi-item validated scale was because questions discerning the desire for an early death were deemed sensitive and a mental health professional was not always available to alleviate any potential distress participants might experience. To create a dummy variable for DTL, we reversed interpretation of the responses for this negatively-phrased question in a way that patients who answered no/not sure were considered to have DTL (DTL = 1). On the contrary, we assigned a value of zero for patients who responded to ever wish to end their life sooner.

#### Physical factors: pain severity

To get a holistic picture of the severity of pain experienced by participants, we elicited patients’ pain experience through four items from Hindi version of the Brief Pain Inventory- Short Form (BPI-SF) [25], at its different levels (worst, least, and on average) and/or in different times (the last 24 hours and the time of responding to the questionnaire). Specifically, participants were asked to rate their pain on a scale of 0 to 10 wherein they can only choose whole numbers (i.e., 0, 1, 2 etc.). We then computed the mean value as a measure of pain severity for patients who responded to at least three out of the four items and left missing otherwise. The number of the latter case is nil. The range of possible values is 0 to 10.

#### Psychological and spiritual factors

##### Psychological distress

We assessed participants’ psychological distress using the Hospital Anxiety and Depression Scale (HADS), which consists of two subscales for measuring anxiety and depression with 7 items each. Each item has 3 levels (1 to 3) with a higher value indicating higher levels of distress (i.e., anxiety or depression). We reverse scored positively phrased statements and summed these item responses to obtain HADS-anxiety and HADS-depression, which ranges from 0 to 21, respectively. For each subscale, HADS defines scores from 0 to 7 as “Normal”, 8–10 as “Borderline Abnormal” or “being suggestive of the presence of anxiety/depression”, and 11–21 as “Abnormal” or “indicative of the probable presence of the mood disorder” [26]. We also combined these two subscales for HADS-total (0 to 42).

##### Self-blame

Participants also answered two questions about how much they blamed themselves for their diagnosis due to their behaviour (e.g., smoking, drinking etc.), which was termed behavioural self-blame (BSB), and due to personal characteristics that they perceive as in-built traits which are not subject to change or susceptible to their control, which was termed characterological self-blame (CSB). Possible responses to these questions were 1 (not at all) to 4 (completely), respectively. For analysis, a dummy variable for the presence of BSB and CSB (BSB = 1 and CSB = 1, respectively) was used as we considered patients who responded with scale 2, 3, or 4 to each question to have experienced either BSB or CSB. This was to ensure an even distribution of patients across the two categories.

##### Spiritual well-being

Using the 12-item Functional Assessment of Chronic Illness Therapy–Spiritual Well-Being Scale (FACIT–Sp) [27, 28], we measured spiritual well-being of participants in two domains: spiritual meaning/peace (from the first 8 items) and faith (from the last 4 items). The former scale measures the patients’ peacefulness, sense of meaning and purpose in life, whereas the latter scale measures their sense of comfort and strength from spiritual beliefs. We reverse scored negatively framed questions and summed item responses so that higher score indicated higher spiritual well-being. Possible scores range from 0 to 32 for the first subscale and from 0 to 16 for the second.

#### Social support

##### Social well-being

The survey instrument included the 7-item Functional Assessment of Cancer Therapy-General questionnaire to assess patients’ social/family well-being (FACT- SWB) [29] through evaluating 1) their communication with and the support provided by their friends, family, partner, and other close relations, and 2) the acceptance of illness by the aforementioned support system (perceived by the patients). Each question asked participants to report levels of support on a scale of 0 (not at all) to 4 (very much). The total score ranges from 0 to 28 with a higher score indicating better social/family well-being.

##### Social stigma

We used the Sense of Stigma factor questions from the Shame Stigma Scale [30] to evaluate feelings of shame or embarrassment patients may have around others’ cognitive and emotional reactions to their cancer diagnosis. Social stigma was scored from 5 (never feels stigma) to 30 (always feels stigma) based on 6 questions which assessed the extent to which patients felt ashamed of, ostracized for, or discriminated against, due to their diagnosis.

#### Awareness of advanced cancer stage

Lastly, we added one categorical-type variable that recorded whether patients were aware of their late cancer stage. Patients were asked if they knew the current stage of their cancer. If they answered with “Advanced Cancer (Stage IV)”, then we assigned a value of one to this variable and zero for otherwise (i.e., “Early Stage (Stage I, II or III) or “I don’t know”).

### Statistical analysis

We analyzed the relationship between patients’ DTL (binary variable: Yes/No) and physical, psychological, spiritual, social factors as well as the awareness of the late cancer stage, using a linear probability regression model. The demographic variables that we controlled for include gender (male = 1, female = 0), age, years of education, a dummy for married (married = 1, otherwise = 0), religion (non-Hindu = 1, Hindu = 0), type of caste (General Caste = 1, otherwise = 0), and SES (poor = 1, otherwise = 0). We used the last variable to identify socially and economically disadvantaged groups of patients whose DTL may be significantly different from that of non-disadvantaged groups. In the survey, patients were asked to report their perceived SES among the four pre-classified options: Poor, Low-Middle class, Upper-Middle class, and Wealthy. We created a dummy variable for the lowest SES, by assigning a value of one for patients reporting “Poor” and zero for others (SES: Poor = 1). One study reports a high correlation between the subjective SES and self-rated health and health outcomes [31]. The self-reported SES may also be a good proxy for financial distress which is often hard to be measured due to large missing data for household income and difficulty in classifying occupational class.

Using linear regression models, we also examined if there are differences in physical, psychological, spiritual, and social factors between a low socio-economic and a high socio-economic group. In the regression models with the dummy for “Poor” SES (SES: Poor = 1) as the primary independent variable of interest, we controlled for other demographic characteristics that include gender, age, education, marital status, caste (general vs. otherwise), and religion (Hindu vs. non-Hindu).

The number of patients for analysis is 192 with no missing information for the variables of interest. An examination of missing data did not reveal any systematic or non-random patterns, and missing scale scores were minimal, ranging across study measure from 1% (on the primary outcome measure, DTL) to 3% (on the stigma measure). All analyses were conducted at STATA 14.

## Results

Table 1 presents the characteristics of the respondents. About 58% of the patients were males. The age of patients ranged from 18 to 81 with a mean of 52 years and the mean years of education was 7 years. Most were married (89%) and Hindu (91%). More than half of the patients fell under disadvantaged caste groups (51%). Also, 23% reported that their perceived SES was “Poor” and 42% reported that they fell under “Lower Middle” class. The most common cancer types were breast (16%), Lung (15%), genitourinary (26%), Oral (8%), and Liver (6%). The proportion of outpatients (60%) exceeded the proportion of inpatients (40%).

**Table 1:** Patient characteristics (*N* = 192)

Panel A: Participant Demographics		
Characteristics		N (%)/ Mean (SD)
Gender, N (%)	Male	112 (58.33%)
	Female	80 (41.67%)
Age, Mean (SD)		51.56 (13.26)
Years of education, Mean (SD)		7.13 (6.32)
Marital Status, N (%)	Married	171 (89.06%)
	Separated	1 (0.52%)
	Widowed	14 (7.29%)
	Divorced	1 (0.52%)
	Never Married	4 (2.08%)
	Missing	1 (0.52%)
Religion, N (%)	Hindu	174 (90.63%)
	Muslim	13 (6.77%)
	Sikh	2 (1.04%)
	Jain	3 (1.56%)
Caste, N (%)	General caste	78 (40.63%)
	Scheduled Caste	18 (9.38%)
	Scheduled Tribe	16 (8.33%)
	Other Backward Class	64 (33.33%)
	Caste: don’t know	10 (5.21%)
	Caste: unknown	6 (3.13%)
Socio-Economic Status (SES), N (%)	Poor	44 (22.92%)
	lower middle	81 (42.19%)
	upper middle	59 (30.73%)
	Wealthy	7 (3.65%)
	SES: unknown	1 (0.52%)
Panel B: Participant QoL Scale Scores		
Characteristics	N (%)/ Mean (SD)	[Min, Max]
Desire to Live (DTL) = 1, N (%)	165 (85.94%)	[0, 1]
BPI- pain severity, Mean (SD)	3.44 (2.26)	[0, 8.25]
Psychological distress, Mean (SD)		
HADS-total	18.70 (7.95)	[0, 38]
HADS-anxiety	8.56 (4.27)	[0, 21]
HADS-depression	10.14 (4.24)	[0, 20]
Presence of Self-blame, N (%)		
Behavioural Self-Blame (BSB) = 1	72 (37.50%)	[0, 1]
Characterological Self-Blame (CSB) = 1	109 (56.77%)	[0, 1]
Spiritual well-being, Mean (SD)		
FACIT-Sp: Total	24.38 (9.14)	[5, 32]
FACIT-Sp: meaning	16.17 (6.05)	[3, 33]
FACIT-Sp: faith	8.22 (4.57)	[0, 16]
Social/Family support (FACT-SWB), Mean (SD)	14.80 (6.15)	[1, 28]
Stigma, Mean (SD)	9.03 (3.55)	[6, 26]
Awareness of late-stage cancer diagnosis = 1, N (%)	45 (23.44%)	[0, 1]

The column titled ‘N (%)/ Mean (SD)’ reports either 1) the number of people that fall in the corresponding category in column ‘Characteristics’ along with their percentage in the sample if the notation ‘N (%)’ is used next to a category or, 2) the mean and standard deviation of the data if the notation ‘Mean (SD)’ is used. (Variable) = 1 notation indicates a binary variable which has a value of 1 when a patient falls in the corresponding category, and a value of 0 otherwise. The following abbreviations are used in the table: i) QoL- Quality of Life ii) DTL- Desire to live iii) SES- Socio-economic status iv) BPI- Brief Pain Inventory v) HADS- Hospital Anxiety and Depression Scale vi) BSB- Behavioural Self-Blame vii) CSB- Characterological Self-Blame viii) FACIT-Sp-Functional Assessment of Chronic Illness Therapy–Spiritual Well-Being Scale ix) FACT-SWB: Functional Assessment of Cancer Therapy-General questionnaire to assess social/family well-being

Patients in our sample reported a mean score of 3.4 (SD: 2.3) for pain severity with the max value of 8.3 out of 10. The mean for HADS-total was 18.7 (SD: 7.9) and the mean scores for HADS-anxiety and HADS-depression were 8.6 (SD: 4.3) and 10.1 (SD: 4.2), respectively. Among the sample patients, 37.5 and 56.8% responded to have behavioral (BSB) and characterological self-blame (CSB), respectively. Patients also reported a mean score of 24.4 (SD: 9.1) out of 48 for spiritual well-being. The mean score for Meaning/Peace subscale was 16.2 (SD: 6.0) and 8.2 (SD: 4.6) for the Faith subscale. The meansocial/family support score was 14.8 (SD: 6.2) out of 28, and the mean social stigma measurement is 9.0 (SD: 3.5) out of 30 with the max value of 26. About 23% patients responded that they were aware of their late-stage cancer diagnosis.

Table 2, Column (1) presents the main results of the linear probability model estimation, showing the associations between patients DTL and their physical, psychological, spiritual, and social factors. We find that pain severity reduced DTL, which is statistically significant. Specifically, one point increase in the BPI pain severity score (ranging from 0 to 10) decreased the probability of having DTL by 2.2%. Among psychological factors, the total score of psychological distress (HADS-total; anxiety & depression, ranging from 0 to 42) is negatively associated with DTL, as we expected. One point rise in the total HADS decreases the probability of having DTL by 0.7%, which is smaller than the effect of pain severity, in absolute sense. On the contrary, we did not find a significant association between the outcome variable and self-blame and social support factors that include the measurement for social and family support and perceived social stigma against cancer and cancer patients. Unlike our hypothesis based on previous literature, spiritual well-being does not significantly affect DTL among our sample patients. We also ran the same regression by disaggregating the total psychological distress and spiritual well-being scores into their sub-scales, respectively. The result is in Column (2), Table 2. The sub-scales of psychological distress (HADS-anxiety and HADS-depression) and spiritual well-being (FACIT-Sp: meaning and FACIT-Sp: faith) are insignificant, possibly due to the lack of statistical power.

**Table 2:** Linear probability regression results of physical, psychological, spiritual, and social factors on Desire to Live (DTL) among advanced cancer patients

	(1)	(2)
	DTL = 1	DTL = 1
BPI- pain severity	−0.022**	−0.022**
	(0.011)	(0.011)
HADS-total	−0.007**	
	(0.003)	
HADS-anxiety		−0.006
		(0.009)
HADS-depression		−0.010
		(0.009)
Behavioural Self- Blame (BSB) = 1	0.029	0.029
	(0.061)	(0.061)
Characterological Self- Blame (CSB) = 1	−0.079	−0.092
	(0.063)	(0.064)
FACIT-Sp: Total	0.002	
	(0.003)	
FACIT-Sp: meaning		−0.004
		(0.006)
FACIT-Sp: faith		0.010
		(0.007)
Social Well Being (FACT-SWB)	0.003	0.003
	(0.005)	(0.005)
Stigma	0.006	0.006
	(0.010)	(0.010)
Awareness of late-stage cancer diagnosis = 1	−0.054	− 0.053
	(0.067)	(0.067)
SES: Poor = 1	−0.162**	−0.158**
	(0.071)	(0.071)
General caste = 1	−0.061	−0.055
	(0.050)	(0.051)
Male = 1	0.005	0.021
	(0.049)	(0.051)
Age	−0.001	−0.002
	(0.002)	(0.002)
Years of education	−0.007	− 0.006
	(0.005)	(0.004)
Married = 1	−0.034	− 0.019
	(0.076)	(0.075)
Non-Hindu = 1	−0.076	− 0.076
	(0.095)	(0.095)
Constant	1.173***	1.238***
	(0.208)	(0.208)
Observations	192	192
R-squared	0.156	0.165

Column (1) reports the main results of the linear probability model estimation, showing the associations between patients DTL (a binary variable) and their physical, psychological, spiritual, and socialfactors. Column (2) reports the same regression results but disaggregating the total psychological distress (HADS) and spiritual well-being (FACIT-Sp) scores into their respective sub-scales‘(Variable) = 1’ notation indicates a binary variable that has a value of 1 when a patient falls in the corresponding category, and 0 otherwise. Robust standard errors are in parentheses. The following abbreviations are used in the table: i) DTL- Desire to live ii) BPI- Brief Pain Inventory iii) HADS- Hospital Anxiety and Depression Scale iv) BSB- Behavioural Self-Blame v) CSB- Characterological Self-Blame vi) FACIT-Sp-Functional Assessment of Chronic Illness Therapy–Spiritual Well-Being Scale vii) FACT-SWB: Functional Assessment of Cancer Therapy-General questionnaire to assess social/family well-being viii) SES- Socio-economic status. *** *p* < 0.01, ** *p* < 0.05, * *p* < 0.1

Only 23% of the 192 sample patients responded that they were aware of their late cancer stage. However, there was no difference in DTL between those who were aware of the late stage and those who were not, when other factors such as pain severity and psychological distress being constant. With regards to demographic information, only SES is significantly associated with DTL. Patients whose perceived SES was poor were likely to have a 16% lower probability of DTL compared to those who had higher SES (lower middle class, upper middle class, and wealthy). By comparing the effects of each variable, we found that SES has the largest effect on DTL.

With the largest effect of SES on DTL in mind, we reported the linear regression results in Table 3 that present whether there are statistically significant differences in patients’ physical, psychological, spiritual, social factors between these two different socio-economic groups. Row (1) (Marginal effect of SES:Poor = 1) reports the coefficients on the dummy variable for the lowest SES (SES:Poor) for each physical, psychological, spiritual, and social factor and row (2) (Mean outcome value among SES:Others = 1) presents the corresponding mean outcome value of the other higher SES groups. Note again that each regression was adjusted for gender, age, years of education, marriage, caste, and religion. We find that only psychological distress (HADS-total) is significantly higher among patients with the lowest SES. When the aforementioned demographic variables are constant, being poor increased the score of psychological distress by 4.1 points. Considering that its mean value of non-poor patients is 17.5, this change is considered to be a large increase because it is equivalent to a 23% increase.

**Table 3:** Linear regression results of an indicator of the lowest SES (SES:Poor = 1) on physical, psychological,and spiritual, and social factors (Outcomes)

Outcomes:	BPI-Pain severity	HADS-total	BSB = 1	CSB = 1	FACIT-Sp: Total	FACT-SWB	Stigma
(1) Marginal effect of SES:Poor = 1	−0.17	4.12***	0.06	0.09	0.18	−0.49	1.18
	(0.43)	(1.58)	(0.09)	(0.09)	(1.81)	(1.24)	(0.86)
(2) Mean outcome value among SES: Others = 1	3.43	17.52	0.36	0.54	24.61	15.20	8.64
*N*	192	192	192	192	192	192	192

Row (1) (Marginal effect of SES:Poor = 1) reports a marginal effect of the lowest SES (poor) on each outcome estimated from the linear regressions. Row (2) (Mean outcome value among SES:Others = 1) reports the corresponding mean outcome values of the other SES group (lower middle class, upper middle class, and wealthy).‘(Variable) = 1’ notation indicates a binary variable that has a value of 1 when a patient falls in the corresponding category, and 0 otherwise. Robust standard errors are in parentheses. All regressions include a dummy for general caste, male dummy, age, years of education, marriage dummy and non-Hindu dummy. The following abbreviations are used in the table: i) BPI- Brief Pain Inventory ii) HADS- Hospital Anxiety and Depression Scale iii) BSB- Behavioural Self-Blame iv) CSB- Characterological Self-Blame v) FACIT-Sp-Functional Assessment of Chronic Illness Therapy–Spiritual Well-Being Scale vi) FACT-SWB: Functional Assessment of Cancer Therapy-General questionnaire to assess social/family well-being vii) SES- Socio-economic status.

*** *p* < 0.01, ** *p* < 0.05, * *p* < 0.1

## Discussion

Using data collected from 192 advanced cancer patients in a tertiary cancer centre in Jaipur, India, this study examines the relationship between various physical, psychological, spiritual, and social factors and desire to live (DTL). Consistent with previous literature [33–35], we found that pain severity reduces DTL. One point increase in the BPI pain severity score decreases DTL by 2.2%. Physical symptoms including pain often bring about feelings of powerlessness and helplessness in patients, which is aggravated by the severity of symptoms and chronicity [36]. For advanced cancer patients in India, living with chronic pain would be particularly more challenging due to limited access to palliative care services, cost of care, and strict regulations on using opioids and other such pain-relieving medication [37].

As a number of earlier studies showed [20, 38–41], this study also found that psychological distress is negatively correlated with DTL. However, its effect size is not as large as the effect size of the pain severity measurement. One point increase in the HADS-total (ranging from 0 to 42) leads to a reduction of DTL by 0.7%. These results contrast to the findings of a study with metastatic cancer patients in Canada that physical pain no longer contributes to the prediction of the desire to hasten death (DHD) once psychological distress variables are included in the model [42]. The authors found that hopelessness and depression accounted for over 34% of the variance in predicting DHD scores. Although our results show a relatively small effect of HADS-total on DTL, the level of psychological distress such as anxiety and depression among the surveyed patients is still worrying. Their average HADS-anxiety and HADS-depression scores were 8.6 and 10.1 respectively, which are not far below the cut-off (11 or higher) for “probable presence of anxiety/depression” [26]. This calls for further attention from healthcare professionals to develop and implement effective strategies to assist patients’ psychological distress management.

Despite its importance, even detecting high psychological distress symptoms is challenging in India. This is because there is a dearth of trained mental health professionals in India [43]. Additionally, both healthcare providers and patients are reluctant to openly talk about this issue [44, 45]. One study shows that healthcare workers are often uncomfortable with managing patients’ extreme emotional reactions [46]. Another study shows that patients find it difficult to disclose depressive symptoms to healthcare providers because they are fearful of being perceived as “less” than others [47]. Patients’ limited understanding of the relationship between psychological distress and physical symptoms could further discourage patients’ willingness to reveal anxiety/depression [48]. More research is needed to clarify what causes patients to hide their psychological distress and to examine the extent to which this behaviour influences the level of actual psychological distress and its effect on DTL.

Holding degrees of pain severity and psychological distress constant, we found socio-economic status had the largest effect on DTL such that patients with perceived socio-economic status “Poor” were likely to have a 16% lower probability of DTL compared to those with higher socio-economic status (i.e., lower middle class, upper middle class and wealthy). We further examined if there are significant differences in patients’ physical, psychological, spiritual, and social factors between the two socio-economic status groups. Controlling for caste, religion, gender, age, marital status and years of education, we found psychological distress to be statistically higher among patients belonging to “Poor” category. Previous work provided similar evidence that cancer patients facing financial difficulties are more likely to feel anxious and depressed, reporting a lower overall quality of life (QoL) [32, 49]. Taken together, our findings imply that it is imperative to improve the QoL of advanced cancer patients from disadvantaged groups by addressing their high psychological distress.

Inconsistent with our hypotheses, we found that self-blame, social support, and spiritual well-being factors have no effect on DTL. In particular, the insignificant effects of social support factors (social well-being and social stigma) are surprising given the prevalence of stigma against cancer patients in a northern state of India [50], very close to where this study was conducted. One possible reason to explain this result could be social desirability: patients might feel the obligation to respond to interviewers in a socially acceptable or desirable manner. This tendency could be salient when the survey questions involve third parties and society,as opposed to questions about how patients feel about themselves. In this case, patients with high social desirability are likely to over-report their social support levels, which leads to measurement errors in the relevant variables.

Lastly, our results show that the awareness of the late cancer stage does not decrease DTL, contributing to the growing evidence that the awareness of diagnosis does not negatively impact patients’ QoL. In several South-East Asian [51–54] and South Asian countries including India, patients’ families often withhold information regarding a cancer diagnosis from the patient, and make treatment decisions on their behalf, believing this exercise protects the patients [55]. Many of them believe that honest and complete disclosure will destroy the patients’ hope and the will to live. However, studies suggest their decision may not be the best one for the patients. One study shows no difference in the prevalence of anxiety and depression between patients who were aware of their diagnosis and those who were not, at an oncology centre in South India [56]. Another study in north India found that the awareness of cancer diagnosis had no significant effect on the QoL of lung cancer patients [57]. In fact, a study with 300 cancer patients at BMCHRC, our study site, found that 94% of them wanted details about their cancer diagnosis and treatment [58]. Combined with these findings, our results lend support to the argument that patients need to be better informed about their diagnosis and prognosis. Yet, this should be carefully interpreted and implemented given the importance of the family’s role in decision making in many South Asian cultures.

Our findings come with the following limitations. First, the data for this study is collected from a single hospital site in India, and hence our findings cannot be generalized for advanced cancer patients in the entire nation. Second, the sampling strategy we used was convenience sampling as opposed to random sampling, which potentially biases the results as people who volunteered to participate in our survey could be different in unobserved ways from the ones who did not. Third, we cannot rule out the possibility of patients giving socially desirable answers, especially for the questions about how other people and the society treat them.

## Conclusions

We examined the relationship between DTL and various physical, psychological, spiritual, and social factors by using data from 192 advanced cancer patients in a tertiary cancer centre in Jaipur, India. We found that pain severity, psychological distress and low socio-economic status reduced DTL of the patients. The negative impact of low socio-economic status is noticeably large in that perceiving oneself as “poor” reduced the probability of DTL by 16%. Considering the limited access to palliative care and high cost of health care in India and other lower middle-income (LMIC) countries, future research should be focused on developing interventions to improve physical pain and psychological distress, particularly for patients who are socially and economically disadvantaged.

### Copyright and licenses

The FACIT and all related works are owned and copyrighted by, and the intellectual property of David Cella, Ph.D. Permission for use of the FACT/FACIT system of questionnaire is obtained by contacting information@facit.org.

BPI-SF© Copyright 1991 Charles S. Cleeland, PhD. Pain Research Group – Used by permission. All rights reserved.

## Data Availability

The datasets used and/or analysed during the current study available from the corresponding author on reasonable request.

## Abbreviations

APPROACH: Asian Patient Perspectives Regarding Oncology Awareness, Care and Health
QoL: Quality of Life
DHD: Desire for Hastened Death
DTL: Desire to Live
SES: Socio-economic Status
HDI: Human Development Index
GDP: Gross Domestic Product
BMCHRC: Bhagwan Mahaveer Cancer Hospital and Research Centre
BPI-SF: Brief Pain Inventory- Short Form
HADS: Hospital Anxiety and Depression Scale
BSB: Behavioural Self-blame
CSB: Characterological Self-blame
FACIT-Sp: Functional Assessment of Chronic Illness Therapy–Spiritual Well-Being Scale
FACT-SWB: Functional Assessment of Cancer Therapy-General questionnaire to assess social/family well-being
LMIC: Lower-Middle Income Countries
